# Computational and Rational Design of Single-Chain Antibody against Tick-Borne Encephalitis Virus for Modifying Its Specificity

**DOI:** 10.3390/v13081494

**Published:** 2021-07-29

**Authors:** Ivan K. Baykov, Pavel Y. Desyukevich, Ekaterina E. Mikhaylova, Olga M. Kurchenko, Nina V. Tikunova

**Affiliations:** Laboratory of Molecular Microbiology, Institute of Chemical Biology and Fundamental Medicine SB RAS, 630090 Novosibirsk, Russia; p.desyukevich@g.nsu.ru (P.Y.D.); e.mikhailova2@g.nsu.ru (E.E.M.); o.kurchenko@g.nsu.ru (O.M.K.)

**Keywords:** antibody, tick-borne encephalitis virus, rational design, computational design, modeling

## Abstract

Tick-borne encephalitis virus (TBEV) causes 5−7 thousand cases of human meningitis and encephalitis annually. The neutralizing and protective antibody ch14D5 is a potential therapeutic agent. This antibody exhibits a high affinity for binding with the D3 domain of the glycoprotein E of the Far Eastern subtype of the virus, but a lower affinity for the D3 domains of the Siberian and European subtypes. In this study, a 2.2-fold increase in the affinity of single-chain antibody sc14D5 to D3 proteins of the Siberian and European subtypes of the virus was achieved using rational design and computational modeling. This improvement can be further enhanced in the case of the bivalent binding of the full-length chimeric antibody containing the identified mutation.

## 1. Introduction

Tick-borne encephalitis virus (TBEV) belongs to tick-transmitted flaviviruses and causes severe arboviral infection in Europe and northeastern Asia [1]. The incidence of TBE has significantly decreased over the past two decades; however, the number of cases remains high. In 2019, more than 3200 cases were officially confirmed in European countries according to the European Centre for Disease Prevention and Control (ECDC) report [2], and approximately 1500 cases were registered in Russia [3]. There are three major subtypes of TBEV, namely, the Far Eastern, Siberian, and European subtypes, each of which differs in terms of severity of the disease [1]. Two new subtypes, Baikalian and Himalayan, have been proposed recently; however, they are rare [4,5]. The mortality caused by the virus depends on the subtype and ranges from 1–2% for the European subtype to 5–20% for the Far Eastern subtype [6]. Long-lasting or chronic neuropsychiatric disorders leading to disability were observed in 10–20% of infected patients [7,8]. Although the Siberian and European subtypes are considered less pathogenic than the Far Eastern subtype, both mild and severe cases of TBE are associated with infection by any of the subtypes [1,9]. In addition, the Siberian subtype has a wide geographical distribution and has been found from Eastern and Southern Europe to the Far Eastern regions [10]. Therefore, all three major subtypes of TBEV require control and prevention.

Currently, there are no approved therapeutic agents to treat TBE [11]. Vaccination is one of the most effective ways to prevent this disease; however, the level of vaccination is insufficient in many European countries and Russia [12,13]. For example, the proportion of vaccinated among people affected by tick bites was only 7–8% in Russia [3,14].

TBEV, like other flaviviruses, has a spherical enveloped virion approximately 50 nm in diameter, containing a positive-sense RNA genome encoding a single polyprotein. TBEV virion contains 180 copies of glycoprotein E, which are organized as homodimers and form a herringbone-like pattern on the surface of the mature virus particle. The virion also contains 180 copies of the M protein. Glycoprotein E is an immunodominant protein and contains three surface domains (D1, D2, and D3, also referred to as DI, DII, and DIII), as well as stem and transmembrane regions [15]. The D3 domain is believed to mediate the binding of the virion to the cell [16,17], which is supported by the fact that the majority of virus neutralizing murine antibodies against flaviviruses are directed to this particular domain [18,19,20,21].

Recombinant antibodies are promising medicines against flaviviruses, including TBEV [15,22,23,24]. The chimeric antibody ch14D5 has been previously constructed and characterized [22]. This antibody binds to the D3 domain of TBEV glycoprotein E (Sofjin-Ru strain) with nanomolar affinity and was shown to provide protection for mice infected with lethal doses of the Absettarov strain of the European subtype of TBEV [22]. Given such prominent antiviral properties, this antibody was considered as a potential therapeutic agent for the prevention and treatment of TBE. It was shown that this antibody has a higher affinity for the recombinant D3 domain of the Far Eastern subtype; however, its affinity for the D3 domains of the Siberian and European subtypes is 1–2 orders of magnitude lower [25]. The affinity of the antibody to the Siberian and European D3 variants can be increased by a rational design. This is supported by the fact that the D3 domains are highly conserved and differ only in positions 313, 317, and 331 for the studied variants (according to flavivirus glycoprotein E designation [26]). Since the neutralizing activity often increases with increasing affinity [27], we believe that the development of antibody variants with a higher affinity for the Siberian and European subtypes will further improve the protective activity of this antibody against these subtypes.

When the spatial structure of the complex is available, rational design and computer modeling methods can be used to increase the affinity of the antibody. There are several approaches that have been successfully used for the rational design of antibodies in order to increase their affinity [28,29,30,31,32,33,34]. In most studies, a positive effect was achieved by the introduction of mutations that either create additional hydrogen bonds or enhance electrostatic or hydrophobic interactions between the antibody and antigen [35,36,37]. The choice of the position for the introduction of mutations, and the choice of mutant residues, is still not straightforward. Thus far, the process has been only partially automated, and human verification of the generated variants and structures was required in most studies [28,29,38]. And even in spite of this, many of the designed variants turned out to be non-functional when tested experimentally [28,32].

In this study, two approaches were used to increase the affinity of the ch14D5 antibody and its single-chain fragment sc14D5 to the D3 domains of the Siberian and European TBEV subtypes: optimization of the geometry of the antigen−antibody interface, and the use of a predictive approach based on machine learning. Optimization of the interface geometry provided the best results and made it possible to increase the affinity to these proteins in comparison with the wild-type antibody sc14D5. We believe that these results are valuable for the development of antibody-based medicines against TBEV.

## 2. Materials and Methods

### 2.1. Design of Mutant Antibody Variants with an Optimized Interface

The design of mutant variants was based on the X-ray structure of the ch14D5 antibody Fab fragment in complex [22] with the D3 domain of the TBEV glycoprotein E, strain Sofjin-Ru [39], obtained with resolution 2.3 Å [40]. Structural analysis and virtual mutagenesis were performed using the UCSF Chimera version 1.13.1. To convert the D3 protein of the Sofjin-Ru strain to the D3 protein of the European subtype within this structure, the Ala331 residue of the D3_Sof protein was replaced by the Thr331 residue rotamer with a chi1 torsion angle equal to minus 61 ° (according to pdb structures 1SVB and 6J5F). Moreover, the Ile317 residue was replaced by the Ala317 residue using the “Rotamers” tool in UCSF Chimera, with the “Dunbrack 2010” rotamer library being selected. The clashes of the Thr331 residue with the antibody residues were analyzed using the “Find Clashes/Contacts” tool in UCSF Chimera, with a van der Waals radius overlap threshold of 0.4 Å.

Then, different variants of amino acid residues were screened using the “Rotamers” tool in UCSF Chimera at positions Tyr_L32 and Tyr_L50 of the antibody light chain, and position Phe_H101 of the antibody heavy chain (sequential numbering starting from the N-termini of the chains). Suitability of substituted amino acid residues was assessed visually, and using the “Find Clashes/Contacts” tool in UCSF Chimera, with a van der Waals radius overlap threshold of 0.4 Å. As a result, mutant residues were selected that would not overlap with adjacent amino acid residues and, at the same time, would be in a fairly probable conformation (within the top half of all possible rotamers for this residue) according to the “Dunbrack 2010” rotamer library.

### 2.2. Predicting the Effect of Antibody Mutations on Binding Affinity Using the mmCSM-AB and mCSM-AB2 Online Services

mmCSM-AB (http://biosig.unimelb.edu.au/mmcsm_ab/ accessed on 10 May 2021) and mCSM-AB2 (http://biosig.unimelb.edu.au/mcsm_ab2/ accessed on 10 May 2021) online services were used to predict the effect of introducing mutations into the Y32M variant of the sc14D5 antibody. A model of the Y32M antibody Fv fragment bound to the D3_Eu protein was used as input data. For this, the regions corresponding to the CH1 and CL constant domains, water molecules and ions were removed from the X-ray structure of the ch14D5 antibody Fab fragment bound to the D3_Sof protein. This X-ray structure was determined previously at 2.3 Å resolution [40]. Thereafter, the Y32M mutation was introduced into the L32 position of the antibody VL domain using UCSF Chimera v. 1.13.1. Similarly, the Ala331 residue of the D3_Sof protein was replaced by the Thr331 residue rotamer with a chi1 torsion angle equal to minus 61°, and the Ile317 residue was replaced by the Ala317 residue. Analysis using the “Antibody design” mode of the mmCSM-AB service resulted in a list of the 100 most favorable triple mutations. The most abundant single mutations were extracted from this list. The individual effect of each of the proposed single mutations was predicted using the “Prediction mode” of the same service. The mCSM-AB2 service was also used to analyze the structure of the Y32M-D3_Eu complex and generate heat maps (Figure S5). The results obtained using the mmCSM-AB and mCSM-AB2 services were almost identical.

### 2.3. Construction of Plasmid DNAs Encoding Single-Chain Antibody Fragments

Sixteen plasmids encoding mutant variants of the sc14D5 antibody were designed in this study. Plasmid DNA pHEN2-14D5 [41] was used as a template for introducing mutations. Mutagenesis was performed by PCR using a high-fidelity Phusion DNA polymerase (Thermo Fisher Scientific, Waltham, MA, USA) and oligonucleotides containing mutations. One part of the target sequence was amplified using the forward flanking primer LMB3_mod_dir (Table S1) and the reverse mutant primer (Table S1), and the second part was amplified using the forward mutant primer and the reverse flanking primer pHEN_mod_rev (Table S1). The resulting DNA fragments were mixed in an amount of 0.5 ng each, and the overlap extension PCR was performed to form a DNA fragment of about 900 bp. The resulting PCR fragments were cleaved with *Nco*I and *Not*I restriction nucleases and ligated to *Nco*I/*Not*I-digested pHEN2-14D5 plasmid. *E. coli* XL1-Blue cells were transformed with the resulting ligation products, plated onto LB-agar with 50 μg/mL ampicillin, and cultivated. After obtaining bacterial clones, DNA isolation and sequencing of the entire single-chain antibody gene using BigDye Terminator v3.1 cycle sequencing kit (Thermo Fisher Scientific, Waltham, MA, USA) was performed.

### 2.4. Production of Single-Chain Antibodies in E. coli

Single-chain antibodies were produced by secretion into the periplasmic space of *E. coli* cells. *E. coli* HB2151 cells transformed with the corresponding plasmid DNA were grown in LB medium (100 μg/mL Amp and 0.1% glucose) at 180 rpm agitation rate at 37 °C. When the optical density OD_600_ reached 0.7–0.9, protein synthesis was induced by adding isopropyl-β-d-1-thiogalactopyranoside (IPTG) to a final concentration of 1 mM; the cultivation was continued at 180 rpm agitation rate at 30 °C. After 6 h, the biomass was separated by centrifugation for 10 min at 6000× *g*. The pellet was resuspended in a buffer containing 20% sucrose and 10 mM Tris-HCl pH 7.5, taken in an amount of 1/10 of the original volume of liquid culture. After incubation for 5 min at room temperature and then 5 min at 0 °C, the cells were pelleted for 2 min at 10,000× *g* at 6 °C. After removing the supernatant, the cell pellet was resuspended in a 5 mM MgSO_4_ solution, taken in an amount of 1/10 of the initial volume of the liquid culture, and incubated for 5 min at 0 °C. Spheroplasts were separated by centrifugation for 2 min at 10,000× *g* at 6 °C, and the supernatant containing periplasmic proteins was used for protein purification.

Single-chain antibodies were isolated from the periplasmic extracts by Ni-NTA chromatography. The periplasmic protein fraction was applied at a flow rate of 1 mL/min to a chromatographic column packed with 1 mL of Ni-NTA agarose (Qiagen, Hilden, Germany). For column washing and elution, phosphate buffered saline (PBS) was used with the addition of NaCl up to 300 mM, containing various concentrations of imidazole. Nonspecifically bound proteins were eluted with a buffer containing 25 mM imidazole, after which the target protein was eluted with a buffer containing 200 mM imidazole. Proteins were concentrated to 2–4 mg/mL concentration using Amicon ultra-4 filters (Millipore, Burlington, MA USA) with a 10 kDa cutoff. The yield of single-chain antibodies was about 0.5 mg from 1 L of bacterial culture.

### 2.5. Production of D3 Proteins in E. coli

The D3_Sof, D3_Zau, and D3_Eu proteins used in this study were fragments of the glycoprotein E of the Far Eastern (strain “Sofjin-Ru”, GenBank number: JN229223.1), Siberian (Zausaev-like strain TBEV-317, GenBank number: MG598844.1), and European TBEV subtypes (GenBank number: KT895092.1), including amino acid residues from 301 to 397 with an additional hexahidstidine tag at the C-terminus. Plasmid DNAs pHEN2-rED3_301 [39], pHEN2-D3_Zau, and pHEN2-D3_Eu [25] were used to produce proteins D3_Sof, D3_Zau, and D3_Eu, respectively. The method for production of D3 domains was similar to that for the single-chain antibodies, except that Amicon ultra-4 filters with a 3 kDa cutoff were used for concentration.

### 2.6. Affinity Constant Measurement

D3 proteins comprising D3 domains of the glycoprotein E were used as antigens, since it was previously shown that the affinity of the ch14D5 antibody for the D3 protein of the Sofjin-Ru strain and for the glycoprotein E of this strain were the same, while D3 protein folded better [39]. The binding of the D3 proteins to antibody mutants was determined by a surface plasmon resonance-based biosensor assay (SPR), using a ProteOn XPR36 system (Bio-Rad Laboratories, Hercules, CA, USA). PBS supplemented with 0.005% Tween-20 (PBST) was used as a system running buffer. The temperature of the sensor chip and autosampler was 25 °C. Antibody screening was performed using D3 proteins covalently immobilized on the surface of one of the vertical channels of the GLH chip (Bio-Rad Laboratories, Hercules, CA, USA) to a 4000–6000 response units (RU) level. The immobilization procedure was performed according to the manufacturer’s recommendations: carboxyl groups of alginate matrix were activated using a mixture containing equal amounts of freshly prepared 10 mM N-hydroxysulfosuccinimide and 40 mM 1-Ethyl-3-(3-dimethylaminopropyl)carbodiimide. Individual solutions of D3 proteins at a 5.5 μg/mL concentration in 10 mM sodium acetate buffer, pH 4.5, were used for immobilization. As soon as desired immobilization level was achieved, unreacted activated carboxyl groups of chip surface were quenched with 1 M ethanolamine-HCl, pH 7.5. To perform screening, periplasmic extracts (45 μL) containing the single-chain antibody were mixed with 195 μL of system buffer and analyzed in the horizontal orientation of the micro channel module (MCM) at a flow rate of 25 μL/min.

Another screening scheme was also used as follows: The analyzed antibodies were immobilized on the surface of the GLH chip to a level of 1000–1500 RU, similar to the process described above. Next, a PBST solution containing one of the D3 proteins with a sufficiently high concentration was passed: 100 nM for the D3_Sof protein and 1000 nM for the D3_Zau or D3_Eu proteins. The results were monitored using the ProteOn Manager 3.1.0 software. The chip surface was regenerated by passing 50 mM NaOH solution for 40 s.

The affinity measurement was carried out as follows: The analyzed antibodies were immobilized on the surface of the GLH chip to a 1000–1500 RU level, similar to the process described above. Serial three-fold dilutions of the D3 proteins in system buffer starting from 3.24 μM concentration were analyzed at a flow rate of 25 μL/min and a horizontal orientation of the MCM. The duration of both the association and dissociation phases was 300 s. D3 protein concentration ranges were determined based on data from scouting experiments. The chip surface was regenerated by passing 50 mM NaOH solution for 40 s. The signal measured at the part of the chip surface where no antibody was immobilized was used as a reference. Global analysis of the experimental data based on a single-site model was performed using the ProteOn Manager v. 3.1.0 software. The measurements were repeated three times, and average dissociation constants (Kd) and their standard deviations (SD) were determined.

## 3. Results

### 3.1. Optimization of the Geometry of the Antigen−Antibody Interface

**Figure 1:**
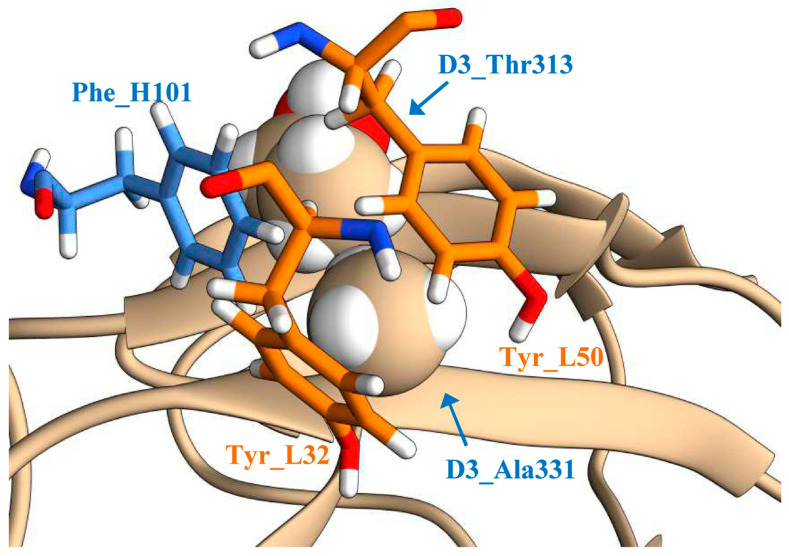
The structure of the antibody−antigen interface adjacent to the Ala331 residue of the D3_Sof protein: The amino acid residues Tyr_L32 and Tyr_L50 of the light chain are shown in orange; the amino acid residue Phe_H101 of the heavy chain of the ch14D5 antibody is shown in blue; part of the D3_Sof protein is shown in tan.

The starting point for the rational design of the antibody was the X-ray structure of the complex of the ch14D5 antibody Fab fragment with the D3 domain of the glycoprotein E of the Far Eastern subtype of TBEV, strain Sofjin-Ru (D3_Sof protein). This X-ray structure was determined previously at 2.3 Å resolution [40]. The analysis of the structure of the antigen−antibody complex showed that the Ala331 residue of the D3_Sof protein is located in a small, hydrophobic pocket on the antibody surface, formed at the contact of the VH and VL domains. The pocket is formed by the aromatic amino acid residues Tyr_L32, Tyr_L50, and Phe_H101 (Figure 1). However, there is not enough room in this pocket for the amino acid residue Thr331, which is located in the same position in the D3 proteins of the Siberian and European TBEV subtypes (D3_Zau and D3_Eu proteins). Increasing the size of this pocket can enhance the affinity between the antibody and the D3_Zau and D3_Eu proteins.

Suitable variants of amino acid substitutions at these positions were determined using structural modeling in the UCSF Chimera version 1.13.1 software. The main criteria for choosing a mutant residue were its size, hydrophilicity/hydrophobicity, and the ability to fit into the context without significant distortion of the geometry of the residue. Variants for which suitable packaging was only achieved in extremely unlikely conformations of the side chain were discarded. Since the OH- group of the Thr331 residue of the D3_Zau protein is likely to be oriented in several different ways, both hydrophilic and more hydrophobic residues were used at positions L32 and H101. A spatial model was generated for each mutant antibody variant (Table 1, Group 1), and it was shown that there was no overlapping of atoms or other structural inconsistencies caused by the introduced substitutions (Figure S1).

To assess the effect of mutations, single-chain antibodies were used. This antibody format was appropriate for this goal because no significant differences were found between the affinity of the ch14D5 antibody Fab fragment to the D3_Sof protein and the affinity of the single-chain variant of the same antibody (sc14D5) to the D3_Sof protein (Figure S2). Furthermore, the use of bacteria-produced proteins made it possible to significantly speed up the process of producing mutant protein variants in comparison with full-length antibodies produced in eukaryotic expression systems. Single-chain antibodies containing the proposed substitutions (Table 1, Group 1) were prepared as purified proteins. Electrophoretic analysis demonstrated that the preparations were highly homogeneous (Figure S3).

**Figure 2:**
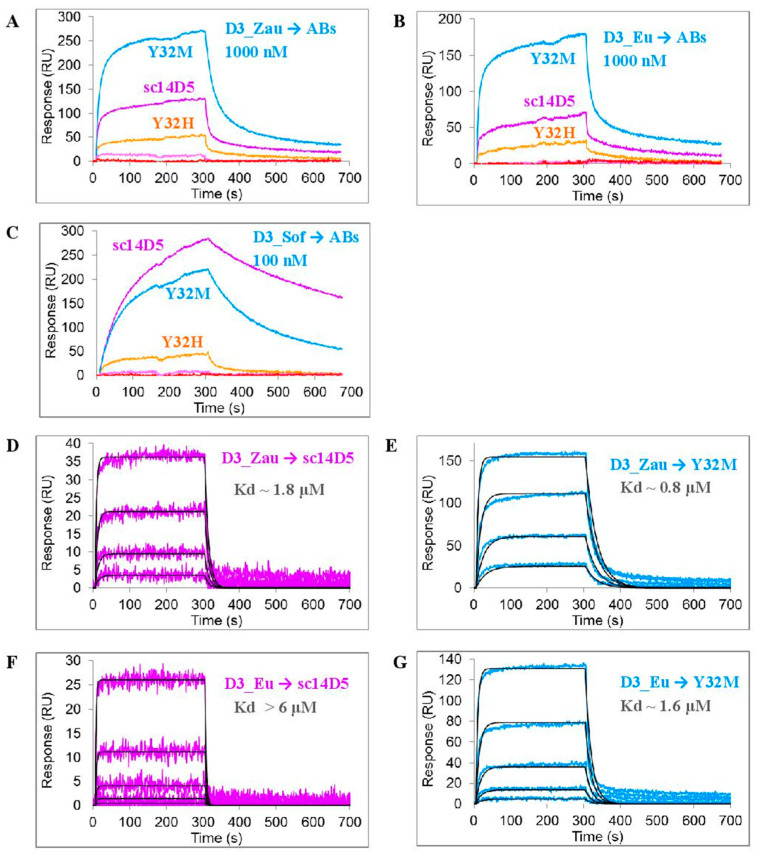
Binding of recombinant D3 proteins to mutant variants of the sc14D5 antibody: (**A–C**) Screening of antibody variants. D3_Zau, D3_Eu, and D3_Sof proteins were used in 1000 nM, 1000 nM, and 100 nM concentrations, respectively. The differences in the concentrations used are due to the differences in the affinity of the studied antibodies with respect to D3 proteins. (**D–G**) Measurement of affinity constants. D3 proteins were used as threefold dilutions starting from 3.24 μM concentration. Experimental curves are shown by colored lines, approximations are shown in black. The analysis was carried out using a ProteOn XPR36 biosensor.

D3 proteins comprising D3 domains of the glycoprotein E were used as antigens. These proteins form a stable immunoglobulin-like fold, which is stabilized by a disulfide bridge [42]. Since it was previously shown that the affinity of the ch14D5 antibody for the D3 protein of the Sofjin-Ru strain and for the glycoprotein E of this strain were the same, D3 proteins were good model antigens [39]. The affinity of the obtained variants of the sc14D5 antibody to the recombinant D3 proteins was measured using the ProteOn XPR 36 biosensor (Bio-Rad Laboratories, Hercules, CA, USA) (Figure 2). Antibody variants were covalently immobilized on the chip surface, and solutions of D3 proteins were passed as an analyte. Since the affinity of the initial antibody ch14D5 for the D3_Eu and D3_ZauT331M proteins (closely related to D3_Zau protein) was 1–2 orders of magnitude lower than for the D3_Sof protein [25], the D3_Eu and D3_Zau proteins were used at a concentration of 1000 nM instead of 100 nM to obtain a sufficiently high signal. At the same time, a 100 nM concentration was sufficient for the D3_Sof protein.

As expected, the affinity of certain mutant variants (namely Y32Q, Y50H, Y50Q, F101L, F101M, and F101Q) to D3 proteins markedly decreased, as no significant binding was observed. However, antibodies Y32M, Y32H, and sc14D5 effectively bound to these proteins. Moreover, the affinity of the Y32M variant for the D3_Zau and D3_Eu proteins was 2.2 times higher than that of the original antibody sc14D5 (Figure 2). This can be seen by the direct measurement of the affinity constants (Figure 2D,G). The range of concentrations used for the D3 proteins in experiments (Figure 2D,E,G) was appropriate because the calculated affinity constants fell within this range. In the case of the interaction of the D3_Eu protein with the sc14D5 antibody (Figure 2F), the calculated affinity constant was outside this range, but the estimate of Kd > 6 μM is sufficient.

### 3.2. Predicting Favorable Mutations using Machine Learning-Based Methods

To further increase the affinity of the constructed Y32M antibody, a machine learning-based method was used to predict the effect of introducing a particular mutation into an antibody. MmCSM-AB was chosen as a computational method [33]. It is available as an online service and uses the spatial structure of the antigen−antibody complex as input data. An X-ray structure-based model of the complex between the Y32M Fv fragment and the D3_Eu protein was used for analysis.

As a result, a set of mutations that could presumably enhance the stability of the antigen−antibody complex was obtained. The most promising mutations were located at positions L_G91, L_G92, H_G100, and H_A102 (Figure S5). Several variants of mutations were chosen for each position (Table 1, Group 2). Structural analysis with the UCSF Chimera was performed to ensure that there was no overlapping of atoms or other structural inconsistencies. It was found that L_G91 position barely accommodates the CH3 group of alanine, while there is no enough space for the more bulky phenylalanine and tryptophan radicals (Figure S4A,B). However, a cavity was identified for the L_G92 position that could be effectively filled with a bulky side chain (Figure S4C). The side chain of the mutant residue at position H_G100 could occupy a rather spacious cavity formed between the D3 domainand the VH and VL domains of the antibody (Figure S4D). However, the H_E99 position seemed unsuitable for substitutions, since this glutamate residue forms a salt bridge with the L_R96 residue of the antibody light chain and K311 lysine of the D3 protein (Figure S4E). Considering that this L_R96 arginine residue is one of the least suitable residues for mutation, as predicted by mmCSM-AB (Figure S5), it was not a good idea to break the H-E99-L_R96 salt bridge. Finally, there was not enough room at position H_A102 to introduce any bulky residues (Figure S4F). However, variants of antibodies with mutated residues L_G91 and H_A102 were not excluded from the study, since it was of interest which of the two methods, i.e., mmCSM-AB or structural assessment in UCSF Chimera, would be more correct.

**Figure 3:**
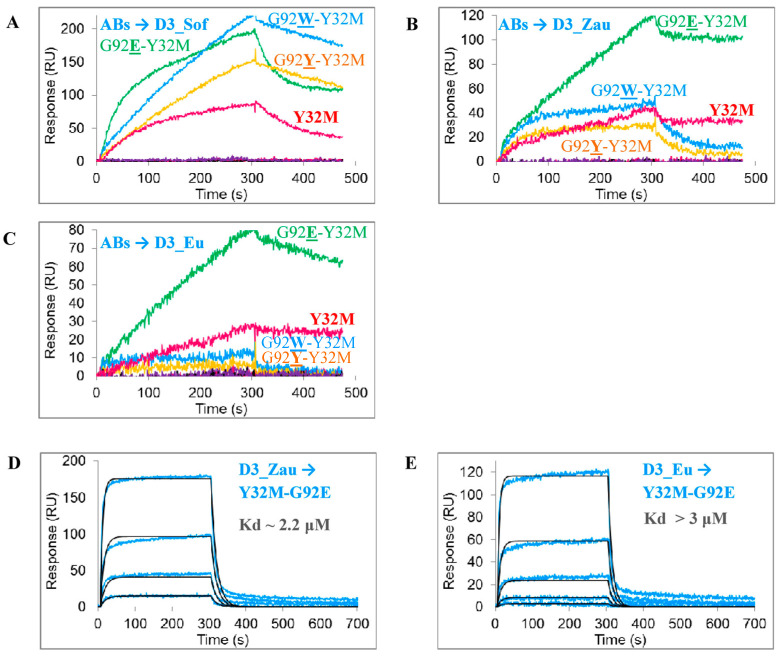
Binding of mutant antibody variants Y32M-G91F, Y32M-G91W, Y32M-G92E, Y32M-G92W, Y32M-G92Y, Y32M-G100W, Y32M-G100Y, and Y32M-A102W to D3 proteins: (**A–C**) *E. coli* periplasmic extracts containing antibody variants were used for screening at approximate antibody concentration of 100–200 nM (as estimated by the intensity of the corresponding bands on the gel electrophoregram). The G92F-Y32M antibody trace is shown in purple. (**D**,**E**) Measurement of affinity constants. Threefold dilutions of purified D3 proteins were used starting from 3.24 μM concentration. Experimental curves are shown by colored lines, approximations are shown in black. The analysis was carried out using a ProteOn XPR36 biosensor.

A screening of the obtained single-chain antibodies showed that the affinity of certain mutant variants of antibodies (namely Y32M-G91F, Y32M-G91W, Y32M-G100W, Y32M-G100Y, and Y32M-A102W) to D3 proteins was markedly decreased as compared to the original antibody sc14D5. However, all variants at the L_G92 position (Y32M-G92E, Y32M-G92W, and Y32M-G92Y) bound to D3 proteins (Figure 3A–C). From these variants, only Y32M-G92E complexes have an off-rate comparable to the Y32M variant (Figure 3B,C, right part of sensorgrams), while the off-rates of the Y32M-G92W and Y32M-G92Y antibodies were much higher due to weaker complexes. Thus, affinity constants were only measured for the Y32M-G92E antibody. The affinity of the purified Y32M-G92E antibody for the D3_Eu and D3_Zau proteins was comparable to the affinity of the original sc14D5 antibody for these proteins (Table 1). Compared to the Y32M antibody, the affinity of the Y32M-G92E antibody was slightly lower (Figure 3D,E, Table 1).

## 4. Discussion

Recombinant antibodies are one of the promising types of medicines against flaviviruses, TBEV in particular [15,22,23,24]. Several antibodies against TBEV have been obtained [43,44,45,46]. Among these antibodies, only a few are virus-neutralizing, and even fewer have a determined sequence. The spatial structure for the antibody−antigen complex has only been determined for 19/1786, Mab 4.2 and T025 antibodies [15,24,47]. However, none of these antibodies have been shown to protect animals against TBEV. The ch14D5 antibody used in this study has a high protective activity, and more pronounced antiviral properties [22,39].

Optimization of the geometry of the antigen−antibody interface and the prediction of stabilizing mutations using a machine learning-based method were used in this study to increase the affinity of the ch14D5-based sc14D5 single-chain antibody for the D3 domains of the Siberian and European TBEV subtypes. The best results were obtained when optimizing the geometry of the antigen−antibody interface. The enlargement of the hydrophobic pocket of the antibody favorably affected the binding of some antibody variants to the D3_Zau and D3_Sof proteins. Of the three positions studied (L32, L50, and H101), only the first position was suitable for introducing substitutions, while mutations at the other two positions strongly destabilized the antigen−antibody complex, which led to a significant decrease in the affinity of these variants. This may be due to the fact that, in the case of the ch14D5 antibody, tyrosine residues L_Y50, L_Y32, and phenylalanine H_F101 form a hydrophobic aromatic core (Figure 1), which stabilizes the interface. This is also confirmed by the fact that these residues are highly conserved in antibodies with similar sequences of the L_CDR2 and H_CDR3 complementarity determining regions. The important role of the H_F101 phenylalanine residue was also shown for other antibodies [35,48]. In addition, analysis using the mmCSM-AB service also predicted that most mutations in the H_F101 residue would lead to destabilization of the complex (Figure S5).

Mutations at the L_Y32 position were generally more favorable, and the Y32M antibody variant had a 2.2-fold higher affinity for the target proteins as compared to the initial single-chain antibody sc14D5. However, the introduction of more hydrophilic histidine or glutamine residues (variants Y32H and Y32Q) was not as successful as the introduction of a methionine residue. The advantage of the methionine residue in this case may be due to its high flexibility and hydrophobicity. Therefore, several other mutant variants containing a flexible and hydrophobic amino acid radical should be tested at position L32 in the future.

A predictive method based on machine learning algorithms was also used to further improve the affinity of the constructed Y32M antibody. The mmCSM-AB method was chosen because it previously showed the highest Pearson correlation coefficient between experimental data and the predicted results [33]. In addition, this method suggested that the introduction of mutations in position H101 would strongly destabilize the antigen−antibody complex (Figure S5), which is in good agreement with the experimental data reporting a strong decrease in the affinity of antibodies F101L, F101M, and F101Q. However, structural analysis of the mutations proposed by the mmCSM-AB method revealed that some of them contradict the geometric requirements. It turned out that there is not enough space in these positions for some of the proposed amino acid residues, and any local displacements in the antibody can lead to the disruption of packaging and destabilization of the interface. Apparently, this is the reason for the significant decrease in affinity for antibody variants containing the L_G91F, L_G91W, or H_A102Y mutations. At the same time, all variants carrying the mutation at position L92 remained capable of binding to D3 proteins; therefore, exhaustive mutagenesis at this position can also lead to increased binding.

Modification of the antibody−antigen interface in the case of the Y32M antibody not only resulted in a 2.2-fold increase in affinity for the D3 proteins of the Siberian and European subtypes, but also in an approximately threefold decrease in the affinity for the D3 protein of the Far Eastern subtype. This was expected. We suggest that the optimized Y32M-based antibody can be used as a cocktail with the original ch14D5 antibody, and that the two antibodies will complement each other in preventing different TBEV subtypes.

To conclude, in this study computational and rational design of the sc14D5 antibody against the glycoprotein E of TBEV was utilized in order to increase the affinity of this antibody to the D3 domains of the Siberian and European TBEV subtypes. A 2.2-fold increase in affinity in the monovalent binding mode can be considered a good result, since the increase in the affinity caused by the introduction of single mutations in similar studies was often of the same order [28,35]. In addition, it is well known that the affinity of full-length antibodies upon bivalent binding is significantly higher than the affinity of monovalent forms of antibodies [49,50,51]. Therefore, on the basis of the achieved results, we can expect a 4–5-fold increase in the affinity of the chimeric antibody ch14D5-Y32M as compared to the original chimeric antibody ch14D5.

## Figures and Tables

**Table 1:** Properties of parental antibodies and constructed mutant variants of the sc14D5 single-chain variable fragments.

Group	Name	Mutation ^1^	Kd (D3_Sof) ± SD	Kd (D3_Zau) ± SD	Kd (D3_Eu) ± SD
Parental antibodies	ch14D5 (bivalently-bound)	No mutations	2.0 ± 0.3 nM	90 ± 20 nM	300 ± 50 nM
	fab_ch14D5		44 ± 6 nM	1.7 ± 0.2 μM	>6 μM
	sc14D5		40 ± 5 nM	1.8 ± 0.2 μM	>6 μM
Group 1 (optimized interface geometry)	Y32H	L_Y32H	+	1.7 ± 0.2μM	>2 μM
	Y32M	L_Y32M	~120 nM	0.80 ± 0.11 μM	1.6 ± 0.2 μM
	Y32Q	L_Y32Q	−	−	−
	Y50H	L_Y50H	−	−	−
	Y50Q	L_Y50Q	−	−	−
	F101L	H_F101L	−	−	−
	F101M	H_F101M	−	−	−
	F101Q	H_F101Q	−	−	−
Group 2 (mmCSM-AB predicted mutations)	Y32M-G91F	L_Y32M + L_G91F	−	−	−
	Y32M-G91W	L_Y32M + L_G91W	−	−	−
	Y32M-G92E	L_Y32M + L_G92E	+	2.2 ± 0.2 μM	>3μM
	Y32M-G92W	L_Y32M + L_G92W	+	+	−
	Y32M-G92Y	L_Y32M + L_G92Y	+	+	−
	Y32M-G100W	L_Y32M + H_G100W	−	−	−
	Y32M-G100Y	L_Y32M + H_G100Y	−	−	−
	Y32M-A102W	L_Y32M + H_A102Y	−	−	−

^1^ The letters H and L indicate whether the mutation belongs to the heavy or light chain of the antibody. “−” indicates that no significant binding was observed; “+” indicates that binding was observed, but no constant was calculated.

## Data Availability

Not applicable.

## Supplementary Materials

The following are available online at www.mdpi.com/article/10.3390/v13081494/s1, Figure S1: Models of complexes for mutant variants of the sc14D5 antibody with the D3_Eu or D3_Zau proteins, Figure S2: Binding of the D3_Sof protein to the Fab-fragment of the ch14D5 antibody (**A**) or the single-chain sc14D5 antibody (**B**), Figure S3: Gel electrophoresis of single-chain antibody fragments in 12% polyacrylamide gel containing SDS, Figure S4: Analysis of the positions predicted using the mmCSM-AB method, Figure S5: Heat map illustrating the effect of additional mutations introduced into the Y32M antibody, predicted using the mCSM-AB2 service, Table S1: Oligonucleotides used in the study.
