# Supplementary material for: Computational and Rational Design of Single-Chain Antibody against Tick-Borne Encephalitis Virus for Modifying Its Specificity
Source: Viruses. 2021 Jul 29;13(8):1494. doi: 10.3390/v13081494 (PMC8402911; doi:10.3390/v13081494)
Supplement: Supplementary file 1 [file viruses-13-01494-s001.zip › Supplementary materials.pdf]

Supplementary materials

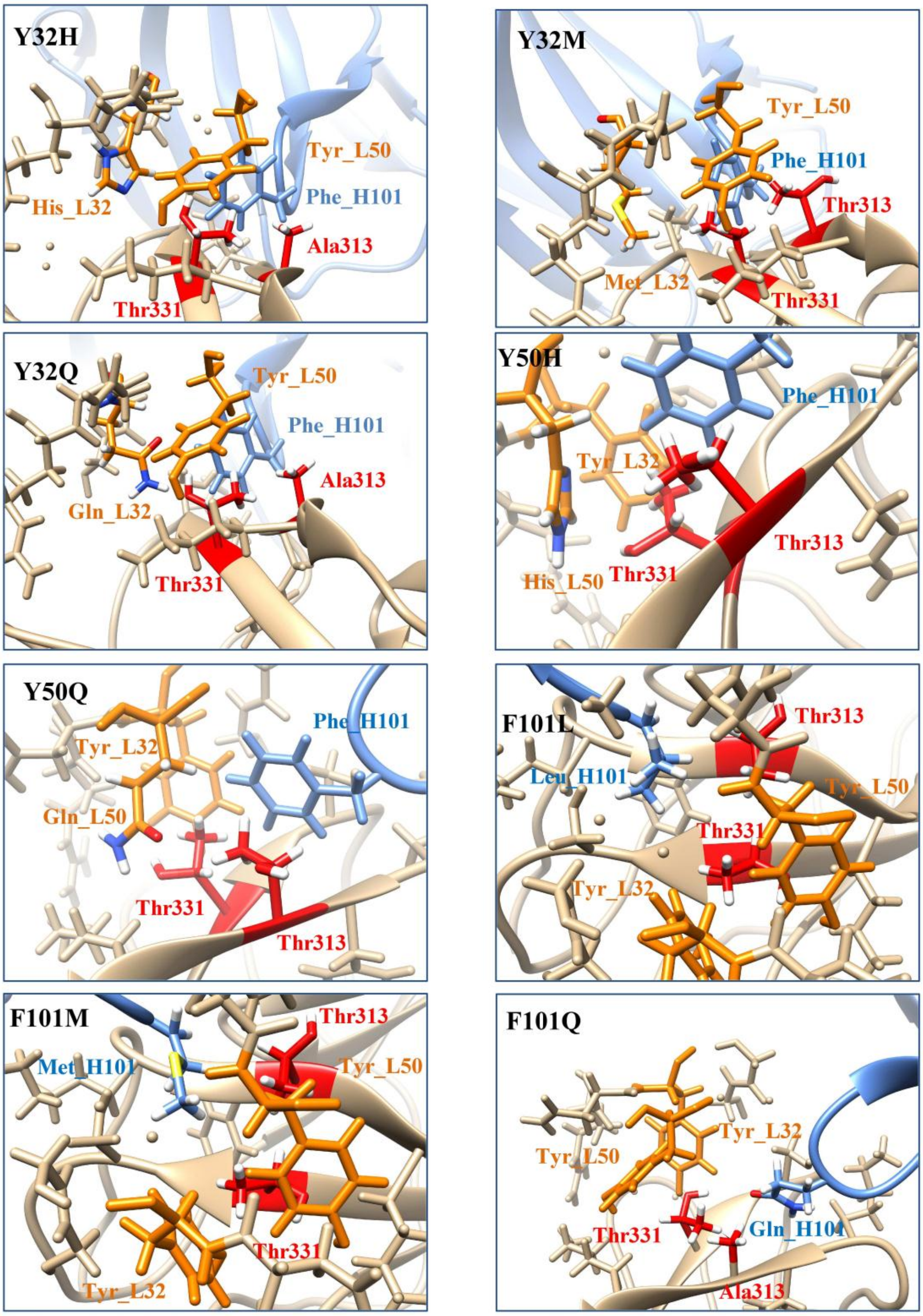

Figure S1. Models of complexes for mutant variants of the sc14D5 antibody with the D3_Eu protein (Y32M, Y50H, Y50Q, F101L, F101M) or D3_Zau (Y32H, Y32Q, F101Q). The amino acid residues of the light or heavy chain are shown in orange or blue, respectively. Part of the D3_Sof protein is shown in tan.

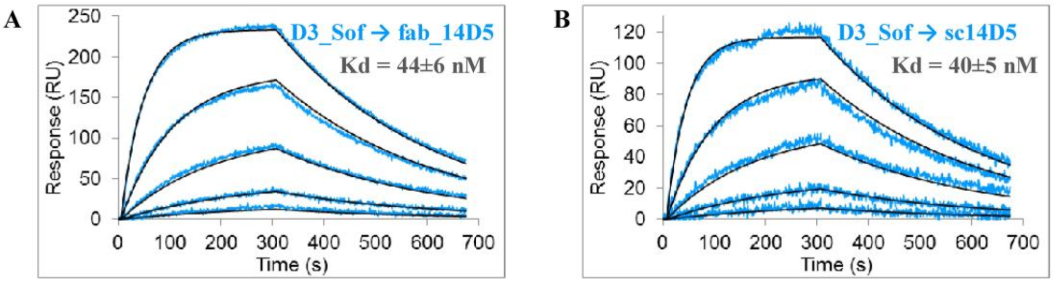

Figure S2. Binding of the D3_Sof protein to the Fab-fragment of the ch14D5 antibody (A) or the single-chain sc14D5 antibody (B). D3 proteins were used as three-fold dilutions starting from 260 nM concentration. Experimental curves are shown in blue, approximations are shown in black.

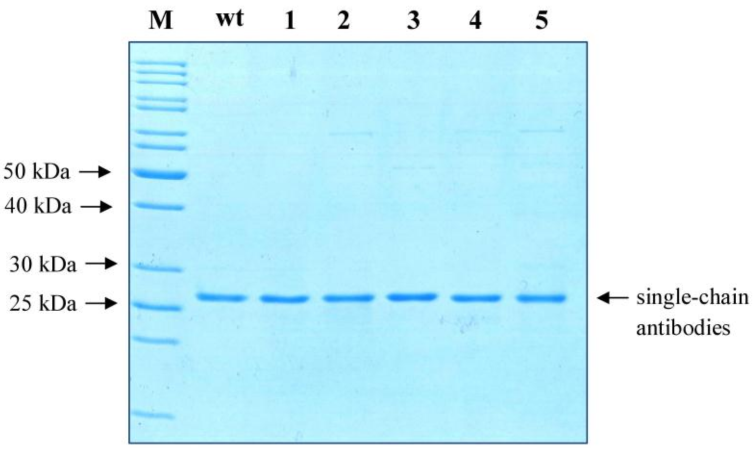

Figure S3. Gel electrophoresis of single-chain antibody fragments in 12% polyacrylamide gel containing SDS. Samples were prepared in non-reducing conditions, 1 μg of proteins were used for analysis. Lane "wt" – single-chain antibody sc14D5, lanes 1-5 correspond to single-chain antibodies Y32H, Y32M, Y50H, F101L and F101M. Other antibody samples looked similar. M – Thermo Fisher scientific protein ladder #26614.

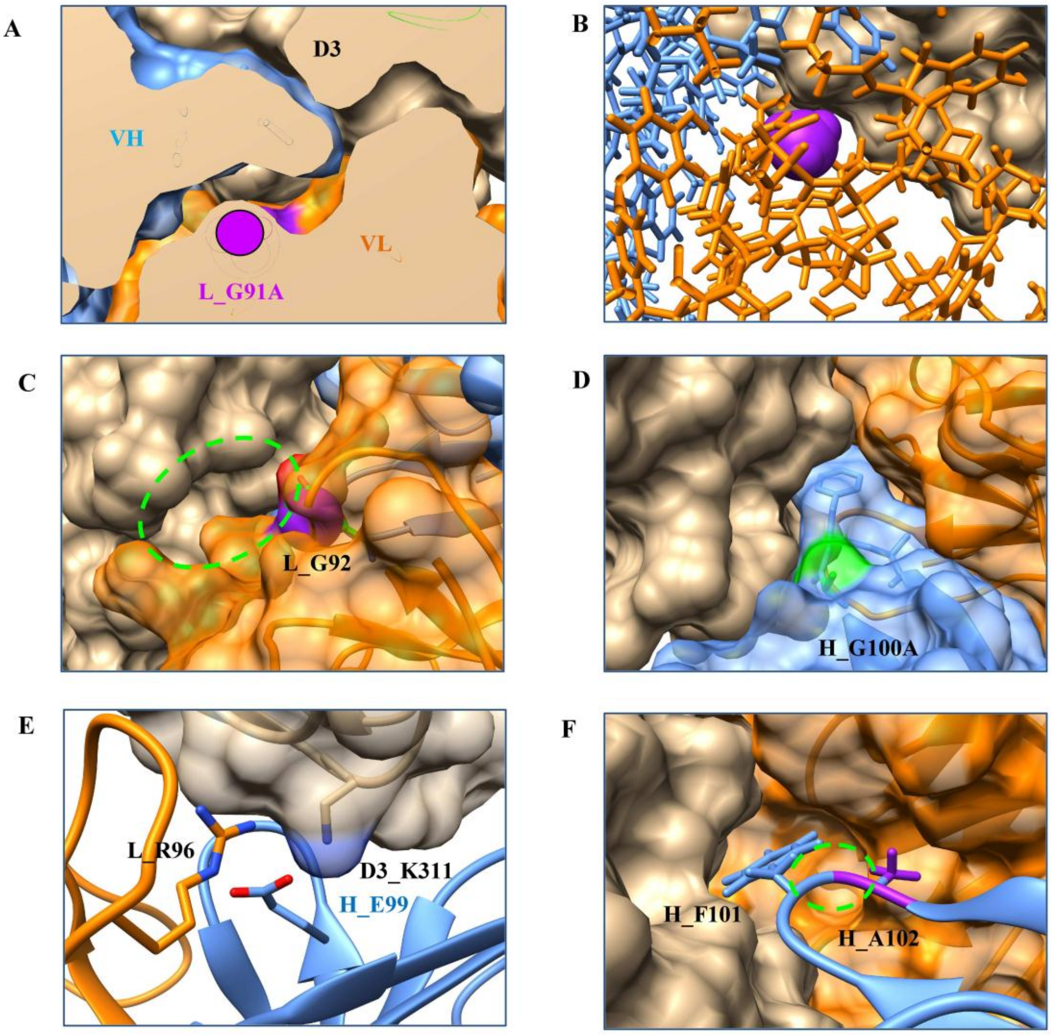

Figure S4. Analysis of the positions predicted using the mmCSM-AB method. (A, B) Analysis of the L_G91 position with introduced alanine mutation. The CH3 group of alanine is shown in purple. (C) Analysis of the L_G92 position. The C-alpha atom of glycine is shown in purple. The cavity between the VL domain and the D3 protein is indicated by the green outline. (D) Analysis of the H_G100 position with introduced alanine residue (shown in green), the CH3 group is directed into the cavity. (E) Analysis of the H_E99 position, the residues involved in the formation of ionic bonds are shown. (F) Analysis of the H_A102 position. A small cavity is outlined in green. Variable domains of antibody are shown in orange or blue, respectively, D3 domain is shown in tan.

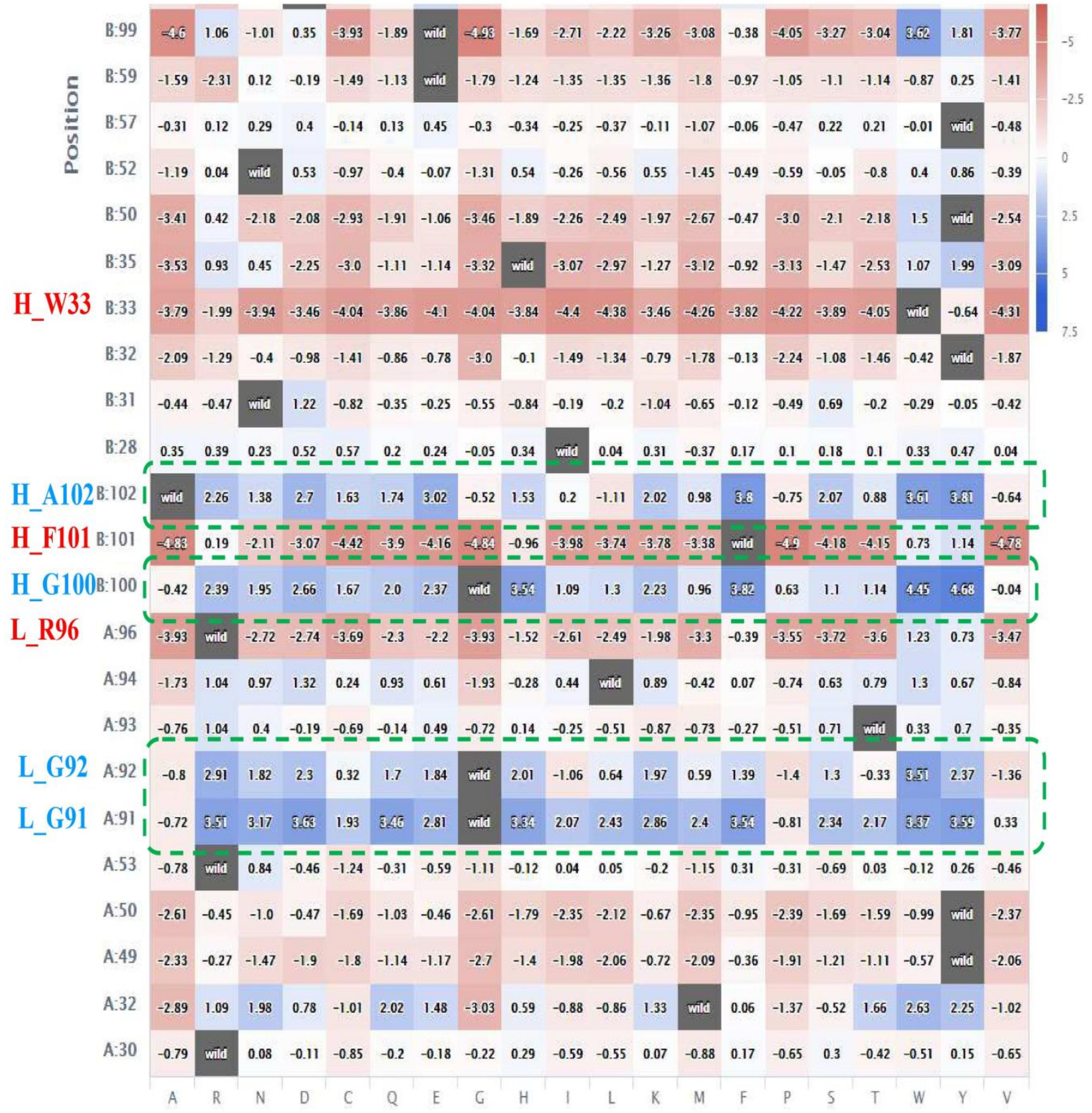

Figure S5. Heat map illustrating the effect of substitutions introduced into the Y32M antibody, predicted using the mCSM-AB2 service (http://biosig.unimelb.edu.au/mcsm_ab2/). The most favorable mutations are shown in blue, the least favorable mutations are shown in red. Amino acid residues of the light chain are marked with "A", the residues of the heavy chain of the antibody are marked with "B". The green outline marks the most favorable positions according to the results of the mCSM-AB2 and mmCSM-AB methods.

Table S1. Oligonucleotides used in the study.

Mutation	Oligonucleotide (5′---3′)
L_Y32H	Y32H_dir: CAGGACATTAGAAATCATTTAAACTGGTATC<br>Y32H_rev: TTGATACCAGTTTAACTGATTTCTAATGTCC
L_Y32M	Y32M_dir: CAGGACATTAGAAATATGTTAAACTGGTATC<br>Y32M_rev: TTGATACCAGTTTAACATATTTCTAATGTCC
L_Y32Q	Y32Q_dir: CAGGACATTAGAAATCAGTTAAACTGGTATC<br>Y32Q_rev: TTGATACCAGTTTAACTGATTTCTAATGTCC
L_Y50H	Y50H_dir: AAACTCCTGATCTACCATACATCAAGATTAC<br>Y50H_rev: CTGTAATCTTGATGTATGGTAGATCAGGAG
L_Y50Q	Y50Q_dir: AAACTCCTGATCTACCAGACATCAAGATTAC<br>Y50Q_rev: CTGTAATCTTGATGTCTGGTAGATCAGGAG
H_F101L	F101L_dir: TGTGTAAGAGAGGGACTGGCCCTTGACTAC<br>F101L_rev: CCAGTAGTCAAGGGCCAGTCCCTCTCTTAC
H_F101M	F101M_dir: TGTGTAAGAGAGGGAATGGCCCTTGACTAC<br>F101M_rev: CCAGTAGTCAAGGGCCATTCCCTCTCTTAC
H_F101Q	F101Q_dir: TGTGTAAGAGAGGGACAGGCCCTTGACTAC<br>F101Q_rev: CCAGTAGTCAAGGGCCTGTCCCTCTCTTAC
L_Y32M_G91F	G91F_dir: TGCCAAGAGTTTGGTACGCTTCCTCGG<br>G91F_rev: CCGAGGAAGCGTACCAAACTCTTGGCA
L_Y32M_G91W	G91W_dir: TGCCAAGAGTGGGGTACGCTTCCTCGG<br>G91W_rev: CCGAGGAAGCGTACCCCACTCTTGGCA
L_Y32M_G92E	G92E_dir: TGCCAAGAGGGTGAAACGCTTCCTCGG<br>G92E_rev: CCGAGGAAGCGTTTCACCCTCTTGGCA
L_Y32M_G92W	G92W_dir: TGCCAAGAGGGTTGGACGCTTCCTCGG<br>G92W_rev: CCGAGGAAGCGTCCAACCCTCTTGGCA
L_Y32M_G92Y	G92Y_dir: TGCCAAGAGGGTTATACGCTTCCTCGG<br>G92Y_rev: CCGAGGAAGCGTATAACCCTCTTGGCA
H_Y32M_G100W	G100W_dir: TGTGTAAGAGAGTGGTTCGCCCTTGAC<br>G100W_rev: GTCAAGGGCGAACCACTCTCTTACACA
H_Y32M_G100Y	G100Y_dir: TGTGTAAGAGAGTATTTCGCCCTTGAC<br>G100Y_rev: GTCAAGGGCGAAATACTCTCTTACACA
H_Y32M_A102Y	A102Y_dir: AGAGAGGGATTCTACCTTGACTACTGG<br>A102Y_rev: CCAGTAGTCAAGGTAGAATCCCTCTCT
Flanking oligonucleotides for insertion into pHEN2	LMB3_mod_dir: TTCACACAGGAAACAGCTATGACC<br>pHEN_mod_rev: CAGTCTATGCGGCCCCATTCA
